# Clinical Characteristics and Oncological Outcomes of Surgically Treated Early-Onset Gastric Adenocarcinoma - a Retrospective Cohort Study

**DOI:** 10.7150/jca.82876

**Published:** 2023-05-21

**Authors:** Ingmar F. Rompen, Henrik Nienhüser, Nerma Crnovrsanin, Julian Musa, Georg Martin Haag, Thomas Longerich, Timon Fiedler, Beat P. Müller-Stich, Leila Sisic, Adrian T. Billeter

**Affiliations:** 1 Department of General, Visceral and Transplantation Surgery, Heidelberg University Hospital, Heidelberg, Germany.; 2 Division of Translational Pediatric Sarcoma Research (B410), German Cancer Research Center (DKFZ) & Hopp-Children's Cancer Center (KiTZ), Heidelberg, Germany.; 3 Department of Medical Oncology, National Center for Tumor Diseases (NCT), Heidelberg University Hospital, Heidelberg, Germany.; 4 Institute of Pathology, Heidelberg University Hospital, Heidelberg, Germany.

**Keywords:** early-onset, young, gastric cancer, adenocarcinoma, surgery

## Abstract

**Introduction:** The incidence of early-onset gastric adenocarcinoma (patients <50 years, EOGA) is rising. Tumors in younger patients are associated with prognostically unfavorable features. The impact of EOGA on patient survival, however, remains unclear. The aim of this study is to evaluate early-onset age as a prognostic factor compared to late-onset gastric adenocarcinoma (LOGA, >50years) in a surgical cohort and assess treatment options.

**Methods:** We analyzed 738 patients (129 early-onset/609 late-onset) operated in curative intent from 2002 to 2021. Data was extracted from a prospectively managed database of an academic tertiary referral hospital. Differences in perioperative as well as oncological outcomes were calculated by chi-square test. Cox regression analysis was performed to assess disease-free survival (DFS) and overall survival (OS).

**Results:** EOGA patients were more often treated with neoadjuvant therapy (62.8% vs. 43.7%, p<0.001) and extended surgical resections e.g. through additional resections (36.4% vs. 26.8%, p=0.027). EOGA was more often metastasized into regional lymph nodes (pN+ 67.4% vs. 55.3%, p=0.012) and to distant sites (pM+: 23.3% vs. 12.0%, p=0.001) and was more often poorly differentiated (G3/G4: 91.1% vs. 67.2%, p<0.001). There were no significant differences in overall complication rates (31.0% vs. 36.6%, p=0.227). Survival analysis showed shorter DFS (median DFS 25.6 months vs. not reached, p=0.006) but similar OS (median OS: 50.5 months vs. not reached, p=0.920) in EOGA compared to LOGA.

**Conclusions:** This analysis confirmed that EOGA is associated with more aggressive tumor characteristics. Early-Onset was not a prognostic factor in the multivariate analysis. EOGA patients may be more capable to undergo intensive multimodal therapy including perioperative chemotherapy and extended surgery.

## Introduction

Gastric adenocarcinoma is the fifth most diagnosed cancer worldwide and has a precarious long-time survival rate of approximately 30% 1, 2. In western countries, incidence is decreasing steadily. However, in the last two decades there has been an increase of 1.2% per year in patients younger than 50 years of age (95%CI:0.9-1.5, p<0.001, 1.5/100'000) now accounting for 7% of all cases 3. Therefore, more possible life years are lost due to cancer related death after the diagnosis of gastric adenocarcinoma. Observations in other gastrointestinal cancer types such as colorectal carcinoma have shown more advanced stages and poorly differentiated cancer in younger patients 4.

Established risk factors for worse oncological outcomes include advanced T-stage, lymph node involvement, metastatic disease, and in some trials also signet ring cell cancers 5. For patients with advanced T-stage and suspected regional lymph node involvement, neoadjuvant treatment with subsequent surgical resection is the standard of care 6, 7. Age is often included in the treatment decisions as a secondary criterion 8. However, due to limited evidence available, age as a criterion for treatment decision is a matter of current debate.

This analysis aims to determine the influence of age on oncological outcomes and asses the differences in clinical phenotypes between EOGA and LOGA patients.

## Methods

This retrospective analysis of a prospectively managed oncological database was performed in compliance with the STROBE guidelines 9. The study was approved by the local ethic committee (Heidelberg University, S-649-2012) and was performed in accordance with the principles of the Declaration of Helsinki and its later amendments.

We reviewed all patients who underwent resection of a gastric adenocarcinoma in curative intent and were operatively treated between 2002 and 2021 at the Department of Surgery of Heidelberg University Hospital, Germany. Exclusion criteria encompassed patients with 1 or multiple of the following criteria; non-resectable tumors undergoing palliative surgery, tumors of the gastro-esophageal junction and above, other active malignancies, and recurrent cancer. In line with the literature, patients below the age of 50 were defined as early-onset and patients older than 50 years of age as late-onset 4.

Treatment decision was made by a multidisciplinary tumor board independent of this study. Treatment included either surgery alone or in combination with (neo-)adjuvant chemotherapy. Diagnostic workup was performed by gastroscopy with tumor biopsy and CT scans of the abdomen and chest. Resection was performed by partial gastrectomy, total gastrectomy, or gastrectomy with transhiatal distal esophagectomy depending on tumor site. Additional resections were subdivided into cytoreduction and hyperthermic intraperitoneal chemotherapy (HIPEC), single organ, and multivisceral resections, which were performed due to infiltrating tumors or metastatic disease. Non-oncologic interventions such as adhesiolysis, cholecystectomies, appendectomies, and hernia repair were not counted as additional interventions in the final analysis. HIPEC was performed due to limited peritoneal carcinosis (peritoneal cancer index <7). Complication rate was calculated from all reported complications regardless of severity. Re-operations included all re-operations due to complications. Two-step approaches due to oncological reasons were excluded from the re-operation rate. Follow-up was conducted yearly until the fifth postoperative year either by clinical consults or by telephone interview. Pathologic workup was conducted using the WHO guidelines, the UICC/AJCC-TNM-Staging 8^th^ Edition (pTNM), and the Lauren classification 10. Poorly differentiated was defined by histologic grade G3 or G4 of pretreatment tumor biopsies. Response to neoadjuvant chemotherapy was categorized according to tumor regression grading by Becker et al. with grade 1a (complete response) and 1b (<10% vital tumor cells) and summarized as adequate histological responders.) 11. Non-partial gastrectomies include total gastrectomies and gastrectomies with transhiatal distal esophagectomy and were compared to partial gastrectomies. Disease-free survival (DFS) was calculated by the difference between time of recurrence and time of surgery. Overall survival (OS) was measured from time of diagnosis to time of death.

### Statistical analysis

Nominal variables were analyzed with chi-square test and ordinal variables were analyzed with the Mann-Whitney U test or Kruskal-Wallis test. Student's T-Test was used to analyze continuous variables with normal distribution and equal variances. Missing data was removed for group comparisons. Univariate survival was estimated using the log rank test and Wald test. The Kaplan-Meier curve was used to demonstrate these findings. The Cox proportional hazards model was used to evaluate associated prognostic factors for DFS and OS. Variables with significant outcomes in the univariate analysis and the variable “early-onset” itself were included in the multivariate analysis. Statistical significancy was defined by a p value <0.05. Analyses were performed in R (v4.2). The computed code and used packages can be found in the supplementary material.

## Results

### Baseline characteristics

A total of 738 patients (129 early-onset and 609 late-onset) met the inclusion criteria. Median age was 43 years (SD 6.41) in EOGA and 67years (SD 9.22) in the LOGA group (p<0.001). Radiologically assessed pretreatment stages (cTNM) were higher within EOGA vs. LOGA and distributed as follows: Stage I 19.7% vs. 29.5%, Stage II 40.2% vs. 47.1%, Stage III 15.7% vs. 11.3% and Stage IV 24.4% vs. 12.1%, p <0.01. Female proportion was higher (51.9% vs. 38.4%, p=0.005, Table 1) and American Society of Anesthesiologists physical status classification system I-VI was lower (ASA 3+4: 37.0% vs. 53.5%, p=0.002) for EOGA compared to LOGA. Patients in the early-onset group were more often treated with neoadjuvant therapy when compared to the late-onset group (62.8% vs. 43.7%, p<0.001 overall and 79.4% vs. 48.6%, p<0.001 since the implementation of standard neo-adjuvant treatment in January 2007). Due to changing evidence in treatment regimen, heterogenous neoadjuvant treatment concepts were used. With 65.7% in EOGA and 73.3% in LOGA, the FLO(T)-regimen with 4 cycles of 5-Fluorouracil, Leucovorin, Oxaliplatin with or without Docetaxel was the most used neo-adjuvant treatment in both groups. Completion of all cycles of neoadjuvant treatment was not significantly different between EOGA and LOGA (95.7% vs. 91.13%, p=0.208). 74.0% of EOGA and 38.8% of LOGA patients received adjuvant chemotherapy (p<0.001). Sites of metastasis of included stage IV patients are presented in Table 2.

### Procedures and oncological quality of surgery

Younger patients underwent more radical local resections while older patients received more partial gastrectomies (non-partial gastrectomy 68.2 vs. 55.5%, p=0.006). Similar rates of R0 resections were achieved in both groups (78.3% vs. 82.1%, p=0.516). The median number of lymph nodes removed was 26 in EOGA and 28 LOGA (p=0.724). Median number of positive lymph.nodes was 1 for EOGA vs. 3.5 for LOGA (p<0.001). Inadequate lymphadenectomy (<16 lymph nodes retrieved according NCCN-guidelines) was found in 11 early-onset (7.9%) and 68 late-onset patients (10.0%, p=0.393).

Additional resections/procedures were performed in 47 EOGA patients and 163 LOGA patients due to infiltrating tumors or metastatic disease (36.4% vs. 26.8%, p=0.027, table 3). 24 (18.6%) EOGA and 104 (17.1%) LOGA patients underwent additional resection of 1 organ/additional tissue (p=0.213) whereas 16 (12.4%) EOGA and 54 (8.9%) LOGA patients had multivisceral resections (p=0.007). Furthermore 8 EOGA and 12 LOGA patients were treated with HIPEC (6% vs. 2.0%, p=0.007). An overview of cancer-affected organs is presented in table 3. Additionally, in 1 patient of both groups the spleen was removed due to intraoperative bleeding.

### Perioperative outcomes

There was no significant difference in complication rate (31.0% vs. 36.6%, p=0.227), Re-operation rate due to complications (15.5% vs. 11.2%, p=0.169) and 30-day mortality (0.0% vs. 1.8%, p=0.151). Anastomotic leakage was reported in 9% of EOGA and 7.4% of LOGA patients (p=0.542). Non-surgical complications such as pneumonia or postoperative cardiac complications occurred less often in EOGA patients than LOGA patients (14.8% vs. 23.1%, p=0.041).

### Pathological characteristics

Comparing the UICC/AJCC-TNM-Staging (pTNM) postoperatively, more EOGA patients were operated with advanced tumor stages (p=0.006, Table 1). When analyzing pTNM separately, the difference in pT stage did not reach statistical significance ([y]pT3/4: 69.0% vs. 62.6%, p=0.168) whereas more advanced lymph node stages ([y]pN+: 67.4% vs. 55.3%, p=0.012) and more metastasized ([y]pM1) tumors were seen in the early-onset group (23.3% vs. 12.0%, p=0.001). Grading of the tumors (UIC-AJCC Grading) showed more poorly differentiated tumors in the early-onset group (G3 or G4: 91.1% vs. 67.2%, p<0.001). In 37 samples, no tumor grading was available. An adequate histological regression after neoadjuvant therapy was equal in both groups (Becker 1a and 1b: 22.9% vs. 23.5%, p=0.480). Signet ring cell positive cancers were more common in the EOGA than in the LOGA group (75.6% vs. 45.8%, p<0.001). In the postoperative histopathological workup of the resected specimens in the EOGA group 70.2% were described as diffuse, 11.6% as mixed-type and 18.2% as intestinal according to the Lauren-classification. In the LOGA group the distribution was 42.9% diffuse-type, 10.5% mixed-type and 45.9% intestinal-type cancers (p<0.001). In 4 LOGA specimens (0.8%) no Lauren-classification was available e.g. due to complete pathological response to neoadjuvant treatment.

### Oncological outcomes

Cancer recurrence was significantly higher within EOGA versus LOGA (50.8% vs. 33.2%, p=0.003). A total of 94.4% of deaths in the early-onset group and 78.2% of deaths in the late-onset group were cancer-related (p=0.024). Median follow up was similar with 40.6 months in both groups.

**Figure 1:**
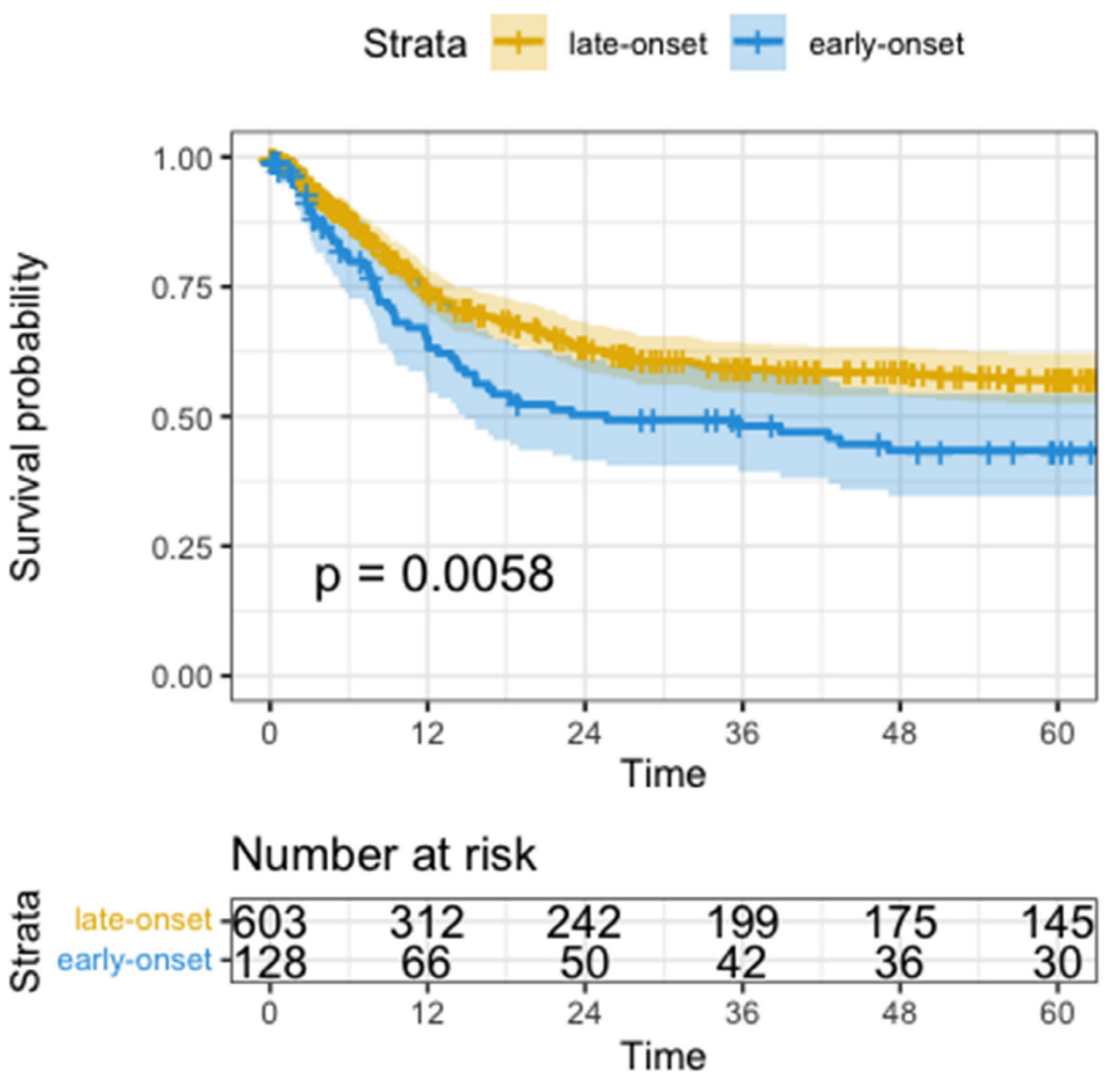
Kaplan-Meyer Curve of Disease-Free Survival. Time in months, p calculated by log-rank test

Median disease-free survival (DFS) was significantly shorter in the early-onset group than in the late-onset group when compared in a univariate analysis by Wald test (median DFS 25.6 months vs. not reached, HR=1.5, 95%CI: 1.1-1.2, p=0.006, Figure 1). Univariate analysis showed significantly worse DFS for receiving neoadjuvant treatment (HR=1.6, 95%CI: 1.3-2.1, p<0.001), total gastrectomy compared to subtotal gastrectomy (HR=1.7, 95%CI: 1.3-2.3, p<0.001), higher UICC-Stage (HR=3.1, 95%CI: 2.7-3.6, p<0.001), higher pT-Stage (HR=2.2, 95%CI: 1.9-2.6, p<0.001), higher pN-Stage (HR=2.0, 95%CI: 1.8-2.2, p<0.001), pM versus non metastatic disease (HR=5.8, 95%CI: 4.4-7.8, p<0.001), R1-Resection versus R0 (HR=3.4, 95%CI: 2.7-4.5, p<0.001), and higher fraction of positive lymph nodes (HR=15.0, 95%CI: 9.9-22.0, p<0.001).

**Figure 2:**
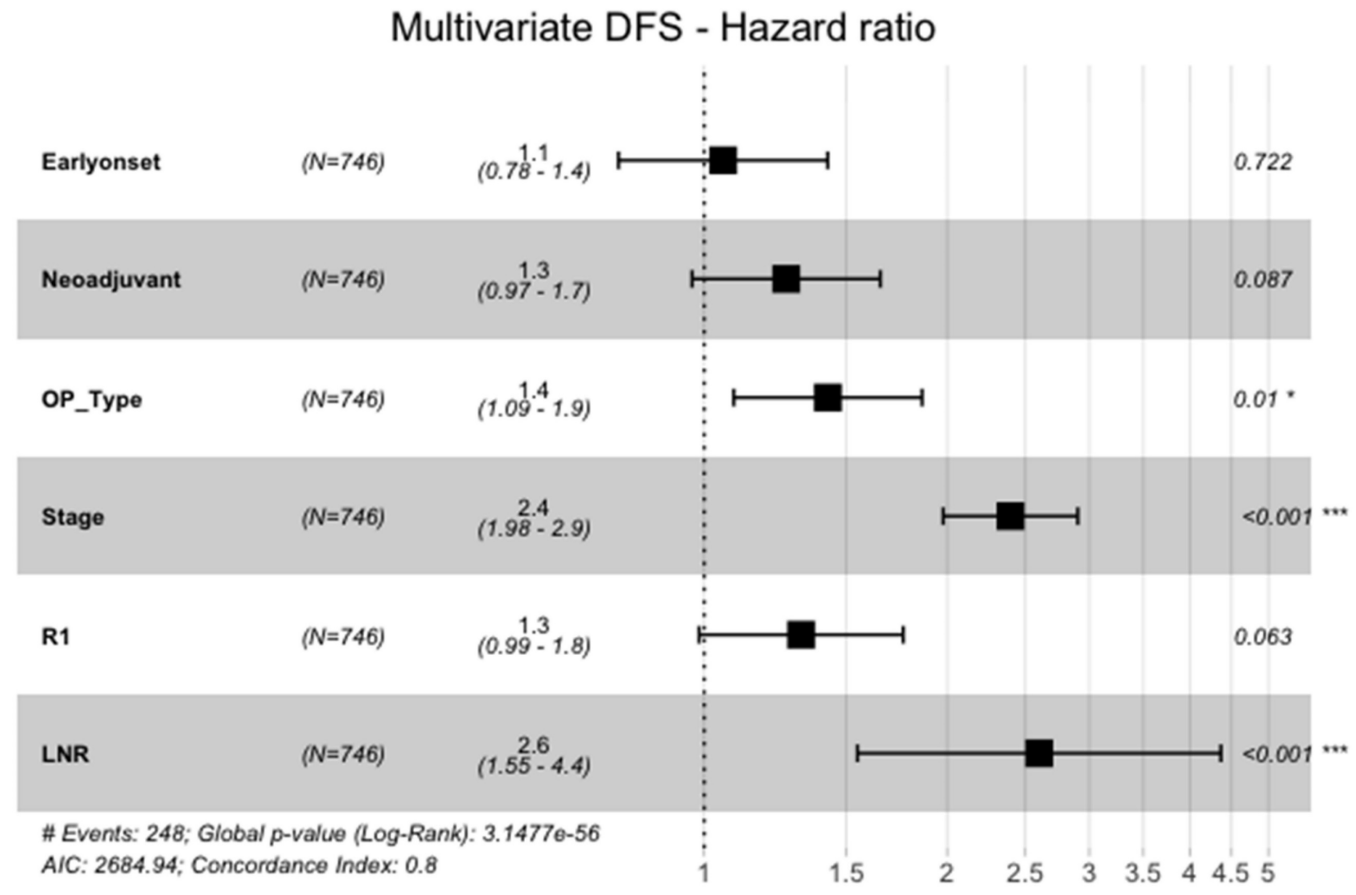
Multivariate Analysis of DFS. Neoadjuvant = neoadjuvant Chemotherapy or Radio-Chemotherapy, OP_Type = subtotal gastrectomy vs. total gastrectomy with or withour transhiatal distal esophagectomy, Stage = UICC-Stages I-IV, LNR = Lymph-Node-Ratio (fraction of lymph nodes with malignant cells)

However, in the multivariate cox-regression model of all significant variables in the univariate analysis, EOGA was not an independent predictor of DFS (HR=1.1, 95%CI: 0.78-1.4, p=0.722, Figure 2). Significant variables for shorter DFS in the cox-regression analysis were advanced UICC stage (HR=2.4, 95%CI: 1.98-2.9, p<0.001), a higher fraction of positive lymph nodes (HR=2.6, 95%CI: 1.5-4.4, p<0.001), and need for total gastrectomy versus partial gastrectomy (HR=1.4, 95%CI: 1.1-1.9, p=0.010).

**Figure 3:**
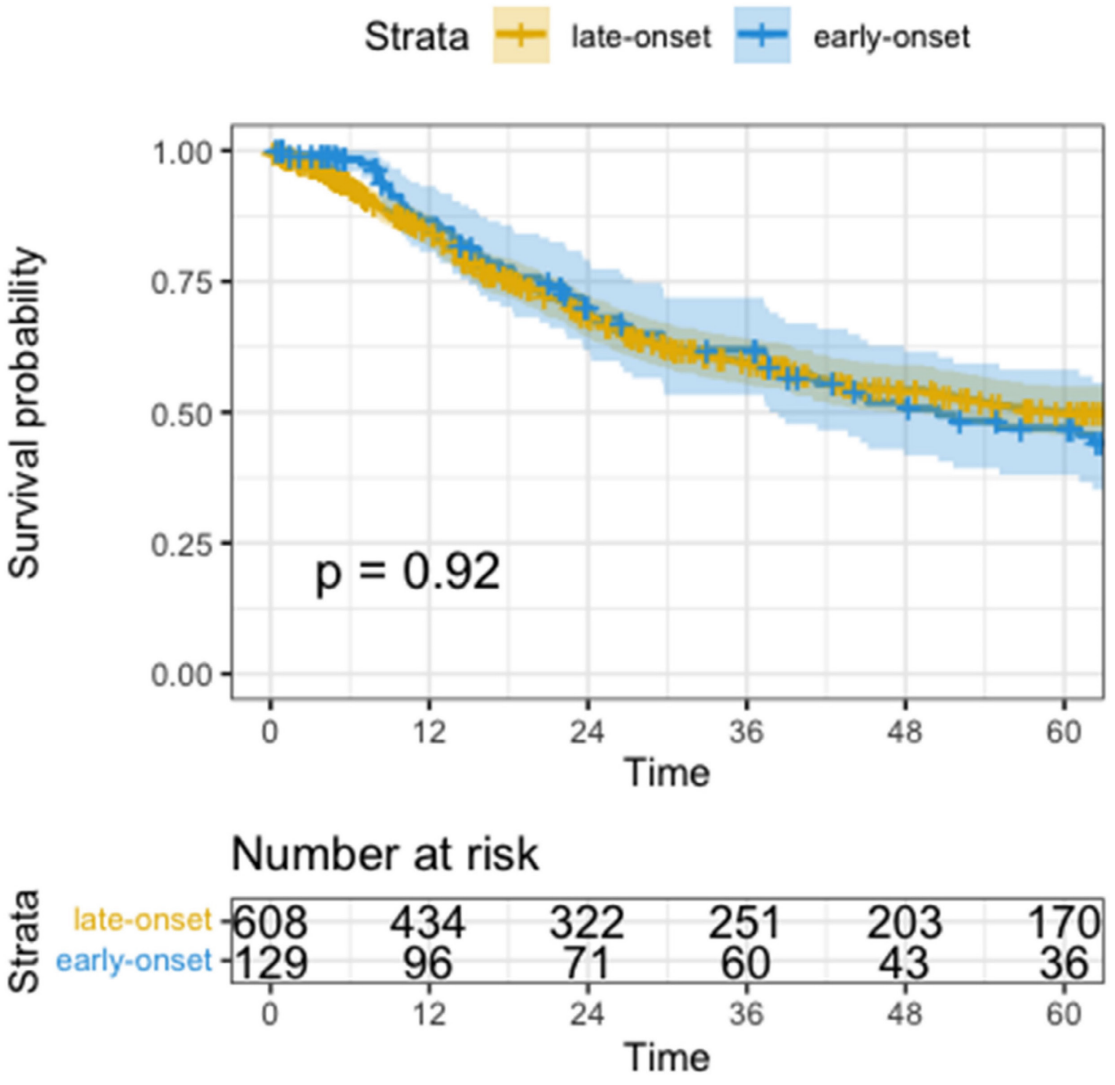
Kaplan-Meyer Curve of Overall survival. Time in months, p calculated by log-rank test

Univariate analysis for overall survival was not significantly different (median OS EOGA 50.5 months vs. LOGA not reached, HR 1.0, 95%CI: 0.7-1.4, p=0.920, Figure 3). Variables that did show worse OS in univariate analysis were higher ASA (HR=1.3, 95%CI: 1.1-1.6, p=0.003), need for total gastrectomy (HR=1.5, 95%CI: 1.2-1.9, p<0.001), having postoperative surgical or medical complications (HR=1.8, 95%CI: 1.4-2.3, p<0.001), advanced UICC-Stage (HR=2.5, 95%CI: 2.2-2.8, p<0.001), higher pT (HR=2.0, 95%CI: 1.7-2.2, p<0.001), higher pN (HR=1.7, 95%CI: 1.6-1.9, p<0.001), pM positive versus non metastatic disease (HR=4.3, 95%CI: 3.3-5.6, p<0.001), R1-Resection versus R0 (HR=3.3, 95%CI: 2.6-4.2, p<0.001) and a higher fraction of positive lymph nodes (HR=14.0, 95%CI: 9.5-20.0, p<0.001).

**Figure 4:**
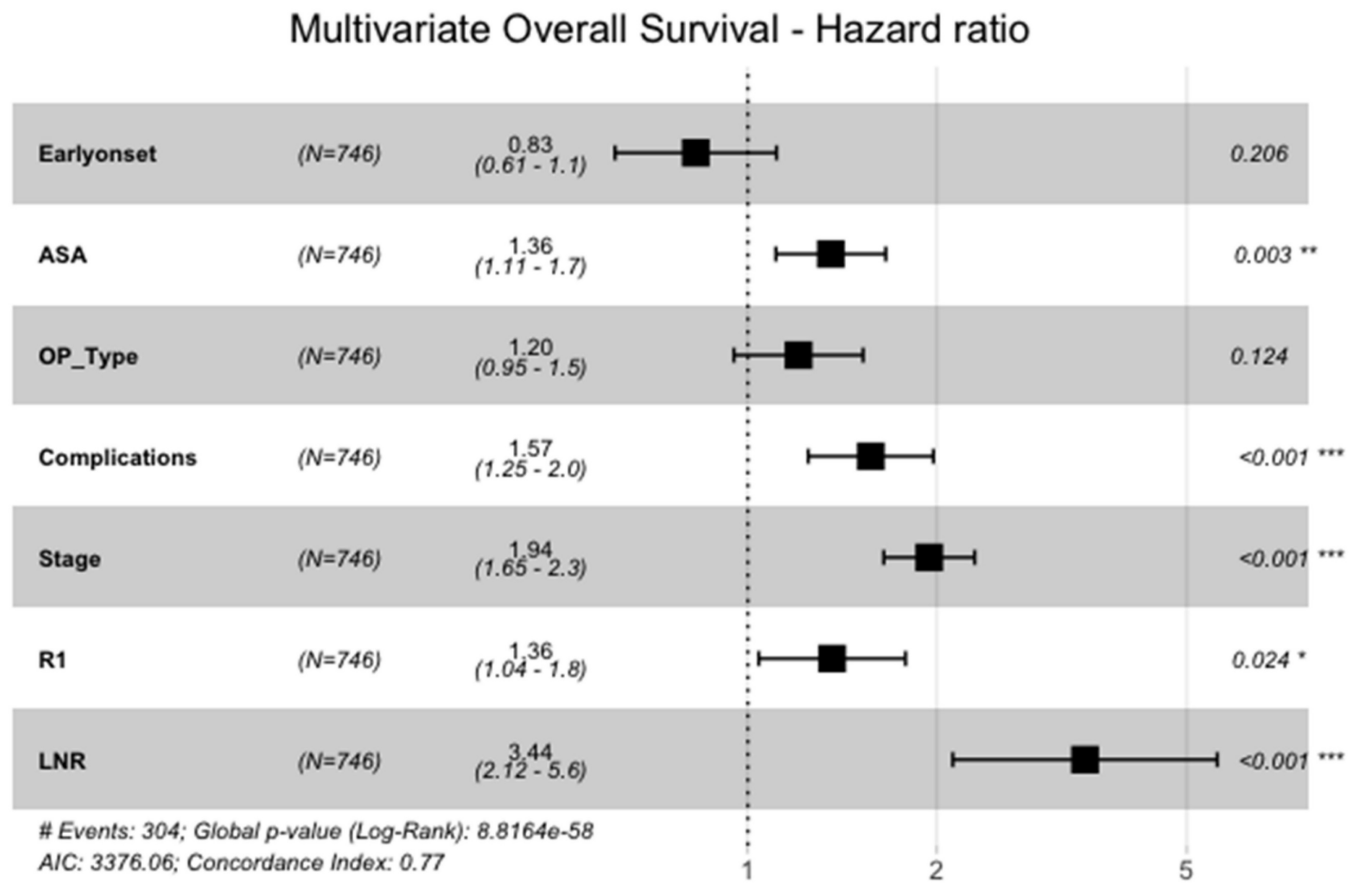
Multivariate Analysis of OS. ASA = American Society of Anesthesiologists physical status classification system I-VI, OP_Type = subtotal gastrectomy vs. total gastrectomy with or withour transhiatal distal esophagectomy, Stage = pUICC-Stages I-IV, LNR = Lymph-Node-Ratio (fraction of lymph nodes with malignant cells)

Also, when analyzed in the multivariate cox-regression model of the above-mentioned significant variables, overall survival of EOGA was not significantly longer than in the late-onset group (HR=0.8, 95%CI: 0.6-1.1, p=0.206, Figure 4). Significant variables for shorter OS were higher ASA (HR=1.4, 95%CI: 1.1-1.7, p=0.003), having perioperative complications (HR=1.6, 95%CI: 1.3-2.0, p<0.001), advanced UICC-tumor stage (HR=1.9, 95%CI: 1.7-2.3, p<0.001), R1-resection versus R0 (HR=1.4, 95%CI: 1.0-1.8, p=0.024) and a higher fraction of positive lymph nodes (HR=3.4, 95%CI: 2.1-5.6, p<0.001).

Cancer-stage adjusted analysis can be seen in Table 1. There was no significant difference for DFS and OS in the stage adjusted analysis between EOGA and LOGA.

### Metastatic disease

A subgroup analysis of 30 EOGA and 73 LOGA patients with synchronous metastasized disease did not show any significant difference in recurrence rate (EOGA 79.3% vs. LOGA 71.8%, p=0.448). Neither median DFS (EOGA 8.0 months SD 0. 4 vs. LOGA 6.8 months SD 0.9, p=0.850) nor median OS (EOGA 20.3 months SD 1. 9 vs. LOGA 13.7 months SD 1.0, p=0.464) showed any significant differences. In both groups peritoneal carcinomatosis was the most common site followed by hepatic metastases (Table 2).

## Discussion

This analysis of 738 patients (129 early-onset and 609 late-onset) represents the largest comparison performed on a surgical cohort of a western population. EOGA patients had more aggressive tumor characteristics such as more advanced stages, more poorly differentiated tumors, and diffuse histology in this analysis. However, EOGA patients were also treated more intensively for example with more extended resections, more multivisceral resections and more neo-adjuvant chemotherapy when compared to LOGA. Despite the aforementioned results, no significant difference in complication rates and overall survival were found.

In line with the analysis of the SEER database, the female to male ratio was higher in the early-onset group. This phenomenon may be partly explained by the difference in exposure to sex hormones in the oncogenesis of diffuse-type gastric cancer 12, 13. In addition, difference in exposure to major risk factors such as smoking, alcohol, and *helicobacter pylori* infection leads to more males in the LOGA group as the effect of environmental carcinogens as well as increasing genetic instability consequently affects majorly elderly patients with longer exposure 14. Hereditary conditions including but not limited to hereditary diffuse gastric cancer (HDGC, CDH1-mutation), Lynch syndrome, Peutz-Jegher and Familial adenomatous polyposis syndrome only account for 1-3% of gastric cancers and therefore only have a limited influence on overall results 15. After review of medical records, only 2 patients were found with hereditary conditions. However, this number may be underestimated as screening was not routinely performed.

In the studied cohort, presence of signet ring cells and tumors with poorly cohesive growth (formerly diffuse histology using the Lauren classification) was significantly higher in the early-onset group 16. This was also concluded in meta-analyses of majorly Asian patients 17, 18. This finding could be related to the fact that poorly cohesive growth in gastric adenocarcinoma is associated with *CDH1*-mutations. In addition to HDGC, analyses of somatic mutations in gastric adenocarcinomas showed a higher frequency of altered *CDH1* in younger patients 19-21, whereas late-onset gastric adenocarcinoma samples did show a higher genomic instability leading to more chromosomal instable tumors and fewer genomically stable tumors (associated with poorly cohesive growth) according to the TCGA molecular classification 19, 22.

In this analysis we found that although early-onset gastric adenocarcinoma is associated with more advanced disease, overall survival rates were not significantly different. Disease-free survival of EOGA, however, is significantly worse in the univariate survival analysis but not in multivariate cox regression analysis. These findings may be explained by more intensive oncologic multimodal treatment of younger patients leading to a prolonged survival 23, 24. For example, EOGA more often received neoadjuvant treatment. Further examples for more intensive treatment are a higher rate of adjuvant treatment applied, additional resections, and intraoperative procedures such as HIPEC in the early-onset group. Moreover, there more extended local resections were performed in the EOGA group. Interestingly, the need for total gastrectomy in some cases with transhiatal distal esophagectomy was associated with worse disease-free survival when compared to partial gastrectomies. This may be influenced by proximal tumor location, more diffuse type tumors, and by lymphatic drainage of the proximal stomach to mediastinal lymph nodes. The limited possibility to resect these lymph nodes during a solely abdominal approach leads to possible minimal residual disease 25. The cox-regression analysis for DFS emphasizes the importance of R0 resections and adequate lymphadenectomy. Due to including non-neoadjuvantly treated patients and Stage IV disease, overall R0-rates in our study were lower than in other studies 6, 26. As shown in the stage-wise analysis (Table 1) and a previous analysis of the same patient cohort, R0 rates are comparable to other studies 6, 26, 27.

More EOGA patients underwent surgical treatment for metastatic disease with metastasectomy and *en-bloc* multiple organ resections (Table 2 & 3). While generally multiorgan resection is associated with more complications, the lower overall complication rate in EOGA and similar overall survival of EOGA and LOGA indicates that such aggressive resections are justified in selected patients 5. Patient selection is crucial since occurrence of complications was associated with shorter overall survival. This may be caused by the complication itself or by the delay of receiving adjuvant therapy 28. As shown in this analysis, younger patients have less comorbidities and may therefore show better tolerance for more intensive chemotherapeutic treatments and extensive surgical resection.

Our study has several limitations. Firstly, this was a retrospective review of a prospectively managed single center surgical database. Secondly, we did not include patients who failed to proceed to surgery. Locally advanced primary tumors with infiltration of adjacent organs, metastatic disease, primary tumor localization, and a higher rate of diffuse type tumors in the EOGA may have influenced decision making for the type of resection and perioperative treatment. Therefore, this analysis cannot conclude if EOGA are truly associated with advanced tumor stages or if the difference is due to extended surgical treatment being offered more frequently to younger patients. Furthermore, the patient cohort of the tertiary referral hospital analyzed may not represent the typical patients in other hospitals. More advanced diseases including patients with oligo-metastasized disease were included in this analysis and had an influence on cancer-related outcomes. The evidence of resections of oligometastatic disease is limited to few retrospective analyses and case reports 29. However, this study supports the resection of oligometastatic disease in selected patients achieiving a median OS of 20 months in Stage IV EOGA. Lastly, the patient population may not be an adequate representation of the world population in terms of sex, age, and other demographic factors. Most studies on EOGA have been performed in Asian populations and are therefore hard to compare due to different tumor biology.

## Conclusion

In this analysis, EOGA is associated with more aggressive tumor characteristics such as advanced tumor stages and poorly cohesive growth. EOGA patients were treated more intensively compared to LOGA including more extended surgical resections. Oncological outcomes showed that overall survival rates were similar, and that early-onset is not a prognostic factor in the multivariate analysis. Further research is needed to assess the role of intensive treatment for gastric adenocarcinoma especially in younger patients who are capable to undergo extended surgical resections.

## Supplementary Material

Supplementary code.

## Figures and Tables

**Table 1:** Stagewise Analysis of pUICC-Stages I-IV

	Overall			Stage I			Stage II			Stage III			Stage IV		
	Early Onset	Late Onset	p	Early Onset	Late Onset	p	Early Onset	Late Onset	p	Early Onset	Late Onset	p	Early Onset	Late Onset	p
Number of Patients	129 (100%)	609 (100%)		28 (21.7%)	182 (29.9%)		28 (21.7%)	145 (23.8%)		43 (33.3%)	209 (34.3%)		30 (23.3%)	73 (12.0%)	
ASA 3/4	47 (37.0%)	323 (53.5%)	**0.002**	8 (28.6%)	93 (51.6%)	0.056	6 (21.4%)	77 (43.5%)	**0.027**	20 (47.6%)	117 (56.2%)	0.704	13 (43.3%)	36 (50.0%)	0.148
Female	67 (51.9%)	234 (38.4%)	**0.005**	11 (39.3%)	63 (34.6%)	0.630	13 (46.4%)	48 (33.1%)	0.177	23 (53.5%)	85 (40.7%)	0.122	20 (66.7%)	38 (52.1%)	0.174
Grade 3/4	112 (91.1%)	388 (67.2%)	**<0.001**	24 (88.9%)	99 (57.9%)	**0.026**	25 (89.3%)	93 (65.5%)	0.068	38 (90.5%)	147 (73.1%)	0.238	25 (96.1%)	49 (84.5%)	**0.036**
Signet ring cells	93 (75.6%)	266 (45.8%)	**<0.001**	20 (71.4%)	72 (41.4%)	**0.012**	22 (81.5%)	55 (39.9%)	**<0.001**	28 (66.7%)	103 (52.3%)	0.089	23 (88.5%)	36 (50.0%)	**<0.001**
Neoadjuvant CTx	81 (62.8%)	266 (43.7%)	**<0.001**	14 (50.0%)	60 (33.0%)	0.079	17 (60.7%)	65 (44.8%)	0.123	27 (62.8%)	104 (49.8%)	0.119	23 (76.7%)	37 (50.7%)	**0.015**
Non-partial Gastrectomy	88 (68.2%)	337 (55.5%)	**0.006**	16 (57.1%)	81 (44.8%)	0.740	19 (67.9%)	81 (55.9%)	0.647	30 (70.0%)	127 (60.7%)	0.201	23 (76.7%)	48 (66.7%)	0.178
Complication Rate	40 (31.0%)	223 (36.6%)	0.227	3 (10.7%)	19 (10.4%)	0.995	7 (25.0%)	45 (31.0%)	0.524	11 (25.6%)	80 (38.3%)	0.114	12 (40.0%)	34 (46.6%)	0.542
Re-OP	20 (15.5%)	68 (11.2%)	0.169	5 (17.9%)	21 (11.5%)	0.345	5 (17.9%)	11 (7.6%)	0.086	3 (7.0%)	22 (10.5%)	0.478	7 (23.3%)	14 (19.2%)	0.634
R0	101 (78.3%)	496 (82.1%)	0.516	28 (100%)	182 (100%)	1.000	28 (100%)	136 (94.4%)	0.202	28 (65.1%)	150 (72.5%)	0.536	17 (56.7%)	28 (39.4%)	0.111
Adequate Regression	19 (22.9%)	65 (23.5 %)	0.480	7 (46.7%)	39 (60.0%)	0.637	4 (23.5%)	13 (19.1%)	0.378	2 (7.1%)	10 (9.3%)	0.771	6 (26.1%)	3 (8.1%)	0.058
Recurrence	61 (50.8%)	190 (33.2%)	**<0.001**	1 (4.5%)	14 (8.1%)	0.556	7 (25.9%)	33 (24.4%)	0.871	30 (71.4%)	97 (49.5%)	0.010	23 (79.3%)	46 (67.6%)	0.246
DFS (median)	25.9 (13.0)	NR	**0.006**	NR	NR	0.442	NR	NR	0.519	13.8 (2.3)	15.4 (2.3)	0.256	8.0 (0.4)	6.8 (0.9)	0.850
OS (median)	50.5 (9.4)	58.9 (9.9)	0.907	NR	NR	0.084	NR	NR	0.061	29.7 (8.0)	24.4 (2.3)	0.965	20.3 (1.9)	13.7 (1.0)	0.464

ASA = American Society of Anesthesiologists physical status classification system I-VI, Non-partial gastrectomies = total gastrectomies with or without transhiatal distal esophagectomy, Neoadjuvant CTx = Neoadjuvant Chemotherapy or Radiochemotherapy, Re-OP = Re-operation, Regression = adequate regression (Becker 1a and 1b), DFS = Disease Free Survival, OS = Overall Survival

**Table 2:** Stage IV patients and Site of Metastasis

	Early Onset Gastric Adenocarcinoma				Late Onset Gastric Adenocarcinoma		
Site of Metastasis	Total	R0-Metastasis	Recurrence	Site of Metastasis	Total	R0-Metastasis	Recurrence
Hepatic	2	2 (100%)	0 (0.0%)	Hepatic	5	3 (60%)	4 (80.0%)
Peritoneal	16	8 (50.0%)	13 (81.3%)	Peritoneal	41	23 (56.1%)	28 (68.3%)
Ovary	1	1 (100%)	1 (100%)	Ovary	3	2 (66.7%)	1 (33.3%)
Lymphatic	3	3 (100%)	3 (100%)	Lymphatic	5	4 (80.0%)	2 (40.0%)
Multiple	7	5 (71.4%)	5 (71.4%)	Multiple	16	5 (31.1%)	7 (43.8%)
Other	1	0 (0.0%)	1 (100%)	Other	3	1 (33.3%)	1 (33.3%)

R0-Metastasis = R0-Resection of the Metastases, Recurrence = Recurrence of gastric cancer any site

**Table 3:** Additional Resections / Procedures

Additional Resections / Procedures	EOGA	%	LOGA	%	p-value
**Total:**	47	36.43%	163	26.77%	0.027
**One Organ/Tissue (partial or total)**	24	18.60%	104	17.08%	0.677
adv. Lymphadenectomy	8	6.20%	18	2.96%	
Liver resections	4	3.10%	20	3.28%	
Colectomy	3	2.33%	18	2.96%	
Pancreatectomy	1	0.78%	15	2.46%	
Splenectomy	2	1.55%	14	2.30%	
Peritonectomy	4	3.10%	11	1.81%	
Adrenalectomy	0	0.00%	4	0.66%	
Nephrectomy	0	0.00%	2	0.33%	
Small bowel	2	1.55%	2	0.33%	
Ovarectomy	1	0.78%	1	0.16%	
Pneumectomy	1	0.78%	0	0.00%	
**Multivisceral (involved organs/tissue)**	16	12.40%	54	8.87%	0.213
Pancreas	12	9.30%	37	6.08%	
Spleen	9	6.98%	32	5.25%	
Colon	6	4.65%	26	4.27%	
Liver	2	1.55%	10	1.64%	
Peritoneum	4	3.10%	8	1.31%	
Kidney	0	0.00%	5	0.82%	
Adrenal gland	3	2.33%	5	0.82%	
Ovaries	2	1.55%	4	0.66%	
**HIPEC**	8	6.20%	12	1.97%	0.007

Adv. Lymphadenectomy = advanced Lyphadenectomy e.g. D3, resecting paraaortal lymph nodes or any other distant lymphadenectomy

## References

[1] Smyth EC, Nilsson M, Grabsch HI, van Grieken NC, Lordick F (2020). Gastric cancer. Lancet.

[2] Ferlay J, Soerjomataram I, Dikshit R, Eser S, Mathers C, Rebelo M (2015). Cancer incidence and mortality worldwide: sources, methods and major patterns in GLOBOCAN 2012. Int J Cancer.

[3] Group (2022). USCSW. U.S. Cancer Statistics Data Visualizations Tool, based on 2021 submission data (1999-2019). US Department of Health and Human Services, Centers for Disease Control and Prevention and National Cancer Institute; wwwcdcgov/cancer/dataviz.

[4] Collaborative R, Zaborowski AM, Abdile A, Adamina M, Aigner F, d'Allens L (2021). Characteristics of Early-Onset vs Late-Onset Colorectal Cancer: A Review. JAMA Surg.

[5] Kulig P, Nowakowski P, Sierzega M, Pach R, Majewska O, Markiewicz A (2021). Analysis of Prognostic Factors Affecting Short-term and Long-term Outcomes of Gastric Cancer Resection. Anticancer Res.

[6] Cunningham D, Allum WH, Stenning SP, Thompson JN, Van de Velde CJ, Nicolson M (2006). Perioperative chemotherapy versus surgery alone for resectable gastroesophageal cancer. N Engl J Med.

[7] Lordick F, Carneiro F, Cascinu S, Fleitas T, Haustermans K, Piessen G (2022). Gastric cancer: ESMO Clinical Practice Guideline for diagnosis, treatment and follow-up. Ann Oncol.

[8] Nienhueser H, Kunzmann R, Sisic L, Blank S, Strowitzk MJ, Bruckner T (2015). Surgery of gastric cancer and esophageal cancer: Does age matter?. J Surg Oncol.

[9] von Elm E, Altman DG, Egger M, Pocock SJ, Gotzsche PC, Vandenbroucke JP (2014). The Strengthening the Reporting of Observational Studies in Epidemiology (STROBE) Statement: guidelines for reporting observational studies. Int J Surg.

[10] Sisic L, Blank S, Nienhuser H, Dorr S, Haag GM, Jager D (2018). Prognostic differences in 8th edition TNM staging of esophagogastric adenocarcinoma after neoadjuvant treatment. Eur J Surg Oncol.

[11] Becker K, Mueller JD, Schulmacher C, Ott K, Fink U, Busch R (2003). Histomorphology and grading of regression in gastric carcinoma treated with neoadjuvant chemotherapy. Cancer.

[12] Kalff MC, Wagner AD, Verhoeven RHA, Lemmens V, van Laarhoven HWM, Gisbertz SS (2022). Sex differences in tumor characteristics, treatment, and outcomes of gastric and esophageal cancer surgery: nationwide cohort data from the Dutch Upper GI Cancer Audit. Gastric Cancer.

[13] Choi Y, Kim N, Kim KW, Jo HH, Park J, Yoon H (2022). Sex-based differences in histology, staging, and prognosis among 2983 gastric cancer surgery patients. World J Gastroenterol.

[14] Milne AN, Sitarz R, Carvalho R, Carneiro F, Offerhaus GJ (2007). Early onset gastric cancer: on the road to unraveling gastric carcinogenesis. Curr Mol Med.

[15] Petrovchich I, Ford JM (2016). Genetic predisposition to gastric cancer. Semin Oncol.

[16] Lauren P (1965). The Two Histological Main Types of Gastric Carcinoma: Diffuse and So-Called Intestinal-Type Carcinoma. An Attempt at a Histo-Clinical Classification. Acta Pathol Microbiol Scand.

[17] Kong X, Wang JL, Chen HM, Fang JY (2012). Comparison of the clinicopathological characteristics of young and elderly patients with gastric carcinoma: a meta analysis. J Surg Oncol.

[18] Niu P, Zhao L, Ling R, Zhao D, Chen Y (2020). Clinicopathological characteristics and survival outcomes of younger patients with gastric cancer: a systematic review and meta-analysis. Transl Cancer Res.

[19] Zhou Q, Tao F, Qiu L, Chen H, Bao H, Wu X (2022). Somatic Alteration Characteristics of Early-Onset Gastric Cancer. J Oncol.

[20] Cho SY, Park JW, Liu Y, Park YS, Kim JH, Yang H (2017). Sporadic Early-Onset Diffuse Gastric Cancers Have High Frequency of Somatic CDH1 Alterations, but Low Frequency of Somatic RHOA Mutations Compared With Late-Onset Cancers. Gastroenterology.

[21] Setia N, Wang CX, Lager A, Maron S, Shroff S, Arndt N (2020). Morphologic and molecular analysis of early-onset gastric cancer. Cancer.

[22] Cancer Genome Atlas Research N (2014). Comprehensive molecular characterization of gastric adenocarcinoma. Nature.

[23] Gong Y, Wang P, Zhu Z, Zhang J, Huang J, Wang T (2020). Benefits of Surgery After Neoadjuvant Intraperitoneal and Systemic Chemotherapy for Gastric Cancer Patients With Peritoneal Metastasis: A Meta-Analysis. J Surg Res.

[24] Martella L, Bertozzi S, Londero AP, Steffan A, De Paoli P, Bertola G (2015). Surgery for Liver Metastases From Gastric Cancer: A Meta-Analysis of Observational Studies. Medicine (Baltimore).

[25] Blank S, Schmidt T, Heger P, Strowitzki MJ, Sisic L, Heger U (2018). Surgical strategies in true adenocarcinoma of the esophagogastric junction (AEG II): thoracoabdominal or abdominal approach?. Gastric Cancer.

[26] Al-Batran SE, Homann N, Pauligk C, Goetze TO, Meiler J, Kasper S (2019). Perioperative chemotherapy with fluorouracil plus leucovorin, oxaliplatin, and docetaxel versus fluorouracil or capecitabine plus cisplatin and epirubicin for locally advanced, resectable gastric or gastro-oesophageal junction adenocarcinoma (FLOT4): a randomised, phase 2/3 trial. Lancet.

[27] Sisic L, Crnovrsanin N, Nienhueser H, Jung JO, Schiefer S, Haag GM (2023). Perioperative chemotherapy with 5-FU, leucovorin, oxaliplatin, and docetaxel (FLOT) for esophagogastric adenocarcinoma: ten years real-life experience from a surgical perspective. Langenbecks Arch Surg.

[28] Lu H, Zhao B, Zhang J, Huang R, Wang Z, Xu H (2020). Does delayed initiation of adjuvant chemotherapy following the curative resection affect the survival outcome of gastric cancer patients: A systematic review and meta-analysis. Eur J Surg Oncol.

[29] Jung JO, Nienhuser H, Schleussner N, Schmidt T (2020). Oligometastatic Gastroesophageal Adenocarcinoma: Molecular Pathophysiology and Current Therapeutic Approach. Int J Mol Sci.

