# Supplementary material for: Clinical Characteristics and Oncological Outcomes of Surgically Treated Early-Onset Gastric Adenocarcinoma - a Retrospective Cohort Study
Source: J Cancer. 2023 May 21;14(9):1470–8. doi: 10.7150/jca.82876 (PMC10266247; doi:10.7150/jca.82876)
Supplement: Supplementary file 1 — Supplementary code. [file jcav14p1470s1.pdf]

Supplementary material of Clinical characteristics and oncological outcomes of surgically treated early onset gastric adenocarcinoma

Rompen et al.

R-code:

```
### survival analyses univariate and cox regression

## name of analysis: early onset gastric adenocarcinoma

# packages
install.packages(c("survival", "survminer"))
install.packages(c("ggplot2", "ggpubr", "dplyr" , "ggfortify"))
install.packages(c("ggfortify"))
library(ggplot2)
library(ggpubr)
library(readxl)
library(survival)
library(survminer)
library(dplyr)
library(ggfortify)


#load data
library(readxl)
filexyz <- read_excel("***")
View(filexyz)


################################univariate
covariates_OS <- c("Earlyonset", "ASA", "Sex",
             "Grade", "Signet_Ring","Neoadjuvant", "OP_Type", "Complications",
             "Stage","pT", "pN","pM", "R1", "Regression", "Timespan", "LNR")
univ_formulas_OS <- sapply(covariates_OS,
                  function(x) as.formula(paste('Surv(OS, Status)~', x)))

univ_models_OS <- lapply(univ_formulas_OS, function(x){coxph(x, data = filexyz)})

# Extract data
univ_results_OS <- lapply(univ_models_OS,
                 function(x){
                  x <- summary(x)
                  p.value<-signif(x$wald["pvalue"], digits=2)
                  wald.test<-signif(x$wald["test"], digits=2)
```

```
                    beta<-signif(x$coef[1], digits=2);#coeficient beta
                    HR <-signif(x$coef[2], digits=2);#exp(beta)
                    HR.confint.lower <- signif(x$conf.int[,"lower .95"], 2)
                    HR.confint.upper <- signif(x$conf.int[,"upper .95"],2)
                    HR <- paste0(HR, " (",
                             HR.confint.lower, "-", HR.confint.upper, ")")
                    res<-c(beta, HR, wald.test, p.value)
                    names(res)<-c("beta", "HR (95% CI for HR)", "wald.test",
                              "p.value")
                    return(res)
                    #return(exp(cbind(coef(x),confint(x))))
                   })

univ_results_OS

## kaplan

model_km_OS <- survfit(Surv(OS, Status) ~ Earlyonset, type="kaplan-meier", data = filexyz)

model_km_OS

# kaplan meier 2

ggsurvplot(
  model_km_OS,
  data = filexyz,
  xlim = c(0, 60),
  break.x.by = 12,
  size = 1,                # change line size
  palette =
    c("#E7B800", "#2E9FDF"),# custom color palettes
  conf.int = TRUE,         # Add confidence interval
  pval = TRUE,             # Add p-value
  risk.table = TRUE,        # Add risk table
  risk.table.col = "BLACK",# Risk table color by groups
  legend.labs =
    c("late-onset", "early-onset"),    # Change legend labels
  risk.table.height = 0.25, # Useful to change when you have multiple groups
  ggtheme = theme_bw()      # Change ggplot2 theme
)

##multivariate
res.cox_OS <- coxph(Surv(OS, Status) ~ +
              Earlyonset+ASA+
                OP_Type+Complications+Stage+
                R1+LNR,
              data =  filexyz)
```

```
summary(res.cox_OS)


## ggforest(res.cox_OS)

ggforest(
  res.cox_OS,
  data = filexyz,
  main = "Multivariate Overall Survival - Hazard ratio",
  cpositions = c(0.02, 0.22, 0.4),
  fontsize = 0.7,
  refLabel = "reference",
  noDigits = 2
)

###DFS

covariates_DFS <- c("Earlyonset", "ASA", "Sex",
                "Grade", "Signet_Ring","Neoadjuvant", "OP_Type", "Complications",
                "Stage","pT", "pN","pM", "R1", "Regression", "LNR", "Timespan")
univ_formulas_DFS <- sapply(covariates_DFS,
                     function(x) as.formula(paste('Surv(NED_op, RezidivProgress)~', x)))

univ_models_DFS <- lapply(univ_formulas_DFS, function(x){coxph(x, data = filexyz)})

# Extract data
univ_results_DFS <- lapply(univ_models_DFS,
                     function(x){
                       x <- summary(x)
                       p.value<-signif(x$wald["pvalue"], digits=2)
                       wald.test<-signif(x$wald["test"], digits=2)
                       beta<-signif(x$coef[1], digits=2);#coeficient beta
                       HR <-signif(x$coef[2], digits=2);#exp(beta)
                       HR.confint.lower <- signif(x$conf.int[,"lower .95"], 2)
                       HR.confint.upper <- signif(x$conf.int[,"upper .95"],2)
                       HR <- paste0(HR, " (",
                                    HR.confint.lower, "-", HR.confint.upper, ")")
                       res<-c(beta, HR, wald.test, p.value)
                       names(res)<-c("beta", "HR (95% CI for HR)", "wald.test",
                                     "p.value")
                       return(res)
                       #return(exp(cbind(coef(x),confint(x))))
                     })

univ_results_DFS

## kaplan
```

```
model_km_DFS <- survfit(Surv(NED_op, RezidivProgress) ~ Earlyonset, type="kaplan-meier",
data = filexyz)

model_km_DFS

# kaplan meier 2

ggsurvplot(
  model_km_DFS,
  data = filexyz,
  xlim = c(0, 60),
  break.x.by = 12,
  size = 1,                # change line size
  palette =
    c("#E7B800", "#2E9FDF"),# custom color palettes
  conf.int = TRUE,         # Add confidence interval
  pval = TRUE,             # Add p-value
  risk.table = TRUE,       # Add risk table
  risk.table.col = "BLACK",# Risk table color by groups
  legend.labs =
    c("late-onset", "early-onset"),    # Change legend labels
  risk.table.height = 0.25, # Useful to change when you have multiple groups
  ggtheme = theme_bw()      # Change ggplot2 theme
)

##multivariate
res.cox_DFS <- coxph(Surv(NED_op, RezidivProgress) ~ Earlyonset+Neoadjuvant+
                     OP_Type+Stage+
                     R1+LNR,
                   data =  filexyz)
summary(res.cox_DFS)


## ggforest(res.cox_DFS)

ggforest(
  res.cox_DFS,
  data = filexyz,
  main = "Multivariate DFS - Hazard ratio",
  cpositions = c(0.02, 0.22, 0.4),
  fontsize = 0.7,
  refLabel = "reference",
  noDigits = 2
)
```
